# Using administrative early-years education data to identify children at risk of persistent absenteeism

**DOI:** 10.23889/ijpds.v9i5.2602

**Published:** 2024-09-18

**Authors:** Megan Wood, Lydia Gunning, Mark Mon-Williams

**Affiliations:** 1 University of Leeds; 2 Born in Bradford's Centre for Applied Education Research

In a post-pandemic world, school absence is an ever-increasing concern for governments, school leaders, and local authorities worldwide. School absence is associated with poor academic outcomes and long-term illness (physical and mental). Absenteeism increases the risk of financial difficulties in adulthood and involvement in the criminal justice system. As a result, the upstream costs to already overstretched public services are crippling. In England, all children are judged by teachers on their “school readiness” over their first year of formal education. We hypothesised that early childhood problems (i.e., not school ready) might be an antecedent of absenteeism. We tested this by investigating the pre-pandemic association between school readiness and persistent absenteeism using a population-linked dataset. Analyses included 62,598 children aged 5-13 years from the Connected Bradford database (spanning academic years 2012/13-2019/20). Special educational needs (SEN) status, English as an Additional Language status, socioeconomic status, sex, and ethnicity were covariates significantly associated with persistent absenteeism. Children who were not “school ready” had increased odds of being persistently absent later in their education journey after controlling for these covariates. School readiness was associated with even greater odds of being persistently absent over two or more years. These findings show: (i) the potential of using existing administrative data to identify children at risk of disengagement from the education system; (ii) absenteeism reflects stark structural inequities. As the rates of absence is still rising, schools can use these data to flag the children who may benefit from early intervention to keep them engaged in school.

